# NT-proBNP as a surrogate for unknown heart failure and its predictive power for peripheral artery disease outcome and phenotype

**DOI:** 10.1038/s41598-023-35073-z

**Published:** 2023-05-17

**Authors:** Bernhard Zierfuss, Anna Feldscher, Clemens Höbaus, Antonia Hannes, Renate Koppensteiner, Gerit-Holger Schernthaner

**Affiliations:** Division of Angiology, Department of Medicine 2, Medical University of Vienna, Waehringer Guertel 18-20, 1090 Vienna, Austria

**Keywords:** Peripheral vascular disease, Heart failure, Risk factors, Prognosis, Outcomes research

## Abstract

Patients with peripheral artery disease (PAD) are at high risk of excess mortality despite major improvements in multimodal pharmacotherapy for cardiovascular disease. However, little is known about co-prevalences and implications for the combination of heart failure (HF) and PAD. Thus, NT-proBNP as a suggested surrogate for HF was evaluated in symptomatic PAD regarding long-term mortality. After approval by the institutional ethics committee a total of 1028 patients with PAD, both with intermittent claudication or critical limb ischemia were included after admission for endovascular repair and were followed up for a median of 4.6 years. Survival information was obtained from central death database queries. During the observation period a total of 336 patients died (calculated annual death rate of 7.1%). NT-proBNP (per one standard deviation increase) was highly associated with outcome in the general cohort in crude (HR 1.86, 95%CI 1.73–2.01) and multivariable-adjusted Cox-regression analyses with all-cause mortality (HR 1.71, 95%CI 1.56–1.89) and CV mortality (HR 1.86, 95% CI 1.55–2.15). Similar HR’s were found in patients with previously documented HF (HR 1.90, 95% CI 1.54–2.38) and without (HR 1.88, 95%CI 1.72–2.05). NT-proBNP levels were independently associated with below-the-knee lesions or multisite target lesions (OR 1.14, 95% CI 1.01–1.30). Our data indicate that increasing NT-proBNP levels are independently associated with long-term mortality in symptomatic PAD patients irrespective of a previously documented HF diagnosis. HF might thus be highly underreported in PAD, especially in patients with the need for below-the-knee revascularization.

## Introduction

Patients with peripheral artery disease (PAD) are at high risk for future cardiovascular (CV) events and mortality^1^. In contemporary registry databases, more than 60% of PAD patients feature atherosclerotic manifestations in other vascular beds^2^. In line with this data, PAD can be regarded as systemic atherosclerotic disease. Despite risk factor control, especially in patients with PAD, the residual CV morbidity and mortality remains high^3–5^. Thus, novel markers for risk stratification in PAD are highly needed.

Despite a lack of accurate evaluations, heart failure (HF) and PAD seem to be common co-prevalent diseases^6,7^. In the EUCLID trial, a randomized controlled comparing clopidogrel vs. ticagrelor in PAD patients, previously known HF was present in about 13% of all included patients. Data of this trial^6^ indicate, that CV morbidity, as evaluated by MACE, and CV mortality were significantly increased by 30–40% in contrast to patients without known HF.

Brain-natriuretic peptide or its N-terminal cleavage product NT-proBNP is a hormone that is predominantly secreted by myocardial tissue in response to volume changes^8^. NT-proBNP was shown to be associated with outcome prediction in heart failure and is a diagnostic marker for heart failure symptoms^9,10^. These results were irrespective of the pathological mechanism of reduced ejection fraction (HFrEF) or preserved ejection fraction (HFpEF)^11^.

Even more, NT-proBNP was capable of outcome prediction in healthy individuals^12–14^, which might partly be explained by undetected heart failure as well. However, in contrast to well-known co-morbidity rates of cerebrovascular disease and coronary heart disease^2^ dedicated reports on prevalence of heart failure are lacking in patients with PAD. Similarly, no study reported NT-proBNPs predictive power for CV-outcome of patients with symptomatic PAD without a diagnosis of heart failure. Encouraged by the current guidelines^15^ on heart failure and strengthened in our focus by the accompanying editorial^16^ we immediately investigated NT-proBNP levels in our patients.

Thus, this study elucidates associations with NT-proBNP in a contemporary cohort of symptomatic PAD and mortality. Additionally, associations between primary stenosis location and NT-proBNP are assessed.

## Results

### Baseline characteristics

A total of 1028 patients were included in the final analysis. For better comparison patients were divided into tertiles of NT-proBNP, as shown in Table 1. Patients of the higher NT-proBNP tertiles were significantly older (p < 0.001), more often of female sex (p < 0.001) but had lower BMI levels (p = 0.043). Rates of type 2 diabetes mellitus (T2DM, p < 0.001) and arterial hypertension (p < 0.001) were higher in the higher tertiles, but active smoking rates (p < 0.001) and rates of hyperlipidemia were lower (p = 0.047). Despite these differences in rates of traditional CV risk factors levels of low-density lipoprotein cholesterol (LDL-C, p = 0.247), HbA1c (p = 0.157) did not differ between the groups, while systolic blood pressure measurements were significantly higher (p < 0.001). Patients with the highest tertile showed higher rates of previous myocardial infarction (p < 0.001), while stroke rates differed only in the lowest vs. the highest tertile with higher rates in the latter (p = 0.015). Patients of the higher tertiles showed worse renal function, as measured by estimated glomerular filtration rate (eGFR, p < 0.001). While similar rates of previous PAD diagnosis (p = 0.318) were seen, rates of critical limb ischemia were significantly higher in the upper tertiles (p < 0.001). Similarly, ankle-brachial index (ABI, p < 0.001) and toe-brachial index (TBI, p < 0.001) measurements were worse in the higher tertiles. All patients had high treatment rates for CV medication but rates for beta-blockers (p < 0.001), calcium channel blockers (p = 0.376), and diuretics (p < 0.001) were higher in the higher tertiles, while renin angiotensin aldosterone system (RAAS)-blockade rates did not differ (0.223). Rates for statin treatment were lower in the higher tertiles (p = 0.003).

**Table 1:** Baseline characteristics.

		1st Tertile	2nd Tertile	3rd Tertile	p-value	1 vs. 3
		(n = 342)	(n = 343)	(n = 343)		
NT-proBNP (pg/ml)		57.1 (38.0, 80.2)	199.3 (147.8, 265.8)	914.2 (575.0, 1847.0)	** < 0.001**	** < 0.001**
Age (years)		62 ± 10	69 ± 10	74 ± 10	** < 0.001**	** < 0.001**
Female sex, n (%)		98 (28.7%)	150 (43.7%)	152 (44.3%)	** < 0.001**	** < 0.001**
Systolic blood pressure (mmHg)		138 ± 17	145 ± 22	155 ± 28	** < 0.001**	** < 0.001**
Diastolic blood pressure (mmHg)		77 ± 10	76 ± 11	79 ± 14	0.353	0.378
BMI (kg/m^2^)		27.4 ± 4.5	26.8 ± 5.2	26.4 ± 5.5	**0.043**	**0.013**
Ankle-brachial-index		0.62 ± 0.16	0.56 ± 0.19	0.54 ± 0.20	** < 0.001**	** < 0.001**
Toe-brachial-index		0.66 ± 0.21	0.58 ± 0.22	0.51 ± 0.19	** < 0.001**	** < 0.001**
History of PAD (%)		106 (31.0%)	110 (32.1%)	124 (36.2%)	0.318	0.153
Fontaine stage (%)	Intermittent claudication	304 (88.9%)	274 (79.9%)	198 (57.7%)	** < 0.001**	** < 0.001**
	Critical limb ischemia	38 (11.1%)	69 (20.1%)	145 (42.3%)		
Active smoking, n (%)		170 (49.7%)	121 (35.3%)	77 (22.4%)	** < 0.001**	** < 0.001**
T2DM, n (%)		124 (36.3%)	140 (40.8%)	171 (49.9%)	**0.001**	** < 0.001**
Art. hypertension, n (%)		288 (84.2%)	311 (90.7%)	324 (94.5%)	** < 0.001**	** < 0.001**
Hyperlipidemia, n (%)		304 (88.9%)	302 (88.0%)	287 (83.7%)	0.095	**0.047**
Previous stroke, n (%)		21 (6.1%)	30 (8.7%)	39 (11.4%)	0.053	**0.015**
Previous myocardial infarction, n (%)		27 (7.9%)	36 (10.5%)	73 (21.3%)	** < 0.001**	** < 0.001**
History of heart failure n (%)		21 (6.1%)	27 (7.9%)	60 (17.5%)	** < 0.001**	** < 0.001**
Serum creatinine (mg/dl)		0.86 (0.75, 1.01)	0.89 (0.75, 1.11)	1.15 (0.88, 1.55)	** < 0.001**	** < 0.001**
eGFR (ml/min/1.73 m^2^)		98.8 ± 19.1	91.3 ± 22.7	71.5 ± 27.0	** < 0.001**	** < 0.001**
LDL-C (mg/dl)		92.1 ± 38.8	88.4 ± 35.5	87.4 ± 37.8	0.247	0.122
HDL-C (mg/dl)		48 ± 16	54 ± 19	50 ± 17	** < 0.001**	0.146
Triglycerides (mg/dl)		133 (100, 193)	122 (90, 169)	122 (90, 170)	**0.013**	**0.009**
HbA1c (rel.%)		6.2 ± 1.1	6.3 ± 1.2	6.4 ± 1.2	0.157	0.056
C-reactive protein (mg/dl)		0.22 (0.12, 0.48)	0.27 (0.10, 0.63)	0.54 (0.19, 1.84)	** < 0.001**	** < 0.001**
Serum total cholesterol (mg/dl)		170 ± 44	170 ± 43	166 ± 46	0.326	0.194
Beta-blockade, n (%)		101 (29.5%)	156 (45.5%)	227 (66.2%)	** < 0.001**	** < 0.001**
RAAS-blockade, n (%)		256 (74.9%)	275 (80.2%)	261 (76.1%)	0.223	0.706
Calcium channel blocker, n (%)		118 (34.5%)	135 (39.4%)	132 (38.5%)	0.376	0.279
Diuretic therapy, n (%)		118 (34.5%)	168 (49.0%)	201 (58.6%)	** < 0.001**	** < 0.001**
Statin therapy, n (%)		314 (91.8%)	310 (90.4%)	288 (84.0%)	**0.003**	**0.002**

Patients are presented in tertiles according to their NT-proBNP level. Data are shown as mean ± standard deviation or median and interquartile range., as applicable. A p-value < 0.05 (two-sided) was considered statistically significant. BMI body mass index. PAD peripheral artery disease, T2DM type 2 diabetes mellitus, eGFR estimated glomerular filtration rate, LDL-C low density lipoprotein cholesterol, HDL-C high density lipoprotein cholesterol, HbA1c hemoglobin A1c, RAAS-blockade renin angiotensin aldosterone system blockade. Bold values in the p-value column depict significant results.

### PAD severity and associations with lesion site

Patients with critical limb ischemia (CLI) had significantly higher NT-proBNP levels than intermittent claudication (IC) patients (log-transformed NT-proBNP: 6.33 ± 1.55 vs 5.12 ± 1.30, p < 0.001). NT-proBNP correlated with worse limb perfusion parameters, as significant inverse correlations between ABI (r = − 0.165, p < 0.001) and TBI (r = − 0.276, p < 0.001) were found.

**Figure 1:**
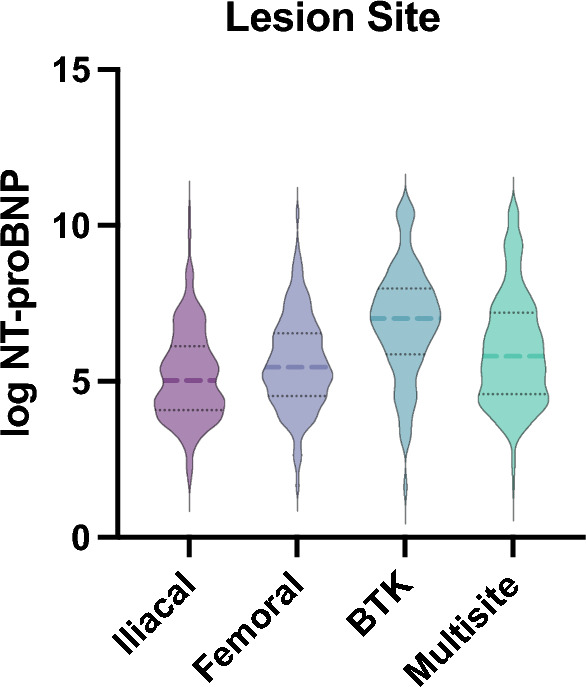
Violin plots for log NT-proBNP shown for four lesion sites (iliacal, femoral, BTK, multisite). ANOVA was significantly different between the groups (p < 0.001) and NT-proBNP levels were the highest in BTK endovascular repair (LDS post-hoc, iliac vs. femoral p = 0.037, iliac vs. BTK p < 0.001, iliac vs. multisite p < 0.001, femoral vs. BTK p < 0.001, femoral vs. multisite p < 0.001, BTK vs. multisite p = 0.003).

Highest NT-proBNP levels were found in patients with target lesions at below-the-knee (BTK) level (6.31 ± 1.72) followed by multisite target lesions (5.69 ± 1.58). The lowest NT-proBNP levels were found in patients with iliac target lesions (5.07 ± 1.39), while SFA (superficial femoral artery)/popliteal lesions were the third highest with 5.32 ± 1.35. A graphical overview can be seen in Fig. 1. ANOVA between the four defined categories was significant (p < 0.001). Post-hoc LSD analyses were significant between all categories (iliac vs. femoral/popliteal p = 0.037, iliac vs. BTK p < 0.001, iliac vs. multisite p < 0.001, femoral/popliteal vs. BTK p < 0.001, femoral/popliteal vs. multisite p < 0.001, BTK vs. multisite p = 0.003).

A binary logistical regression model for NT-proBNP and stenosis location with iliac and femoral/popliteal vs. BTK and multisite stenosis was performed to adjust for site-specific risk factors. Both univariate (OR 1.29, 95% CI 1.17–1.43) and multivariable-adjusted (age, sex, LDL-C, active smoking, HbA1c, arterial hypertension, eGFR and Fontaine stage) binary logistic regression analyses showed significant associations for each increase of one-unit of logarithmically transformed NT-proBNP increase (OR 1.14, 95% CI 1.01–1.30). An overview is presented in Table 2.

**Table 2:** Binary logistic regression analysis for iliacal or femoral target lesions vs. below-the-knee and multitarget site lesions.

Binary logistic regression analyses			
	OR	95% CI	p-value
Univariate	1.29	1.17–1.43	** < 0.001**
Multivariable adjusted	1.14	1.01–1.30	**0.049**

Data are presented as odds ratio and 95% confidence interval for an increase of one-unit of logarithmically transformed NT-proBNP levels. Models were adjusted for age, sex, LDL-C, active smoking, HbA1c, arterial hypertension, eGFR and Fontaine stage.

Significant values are presented in bold.

### Outcome analyses

**Figure 2:**
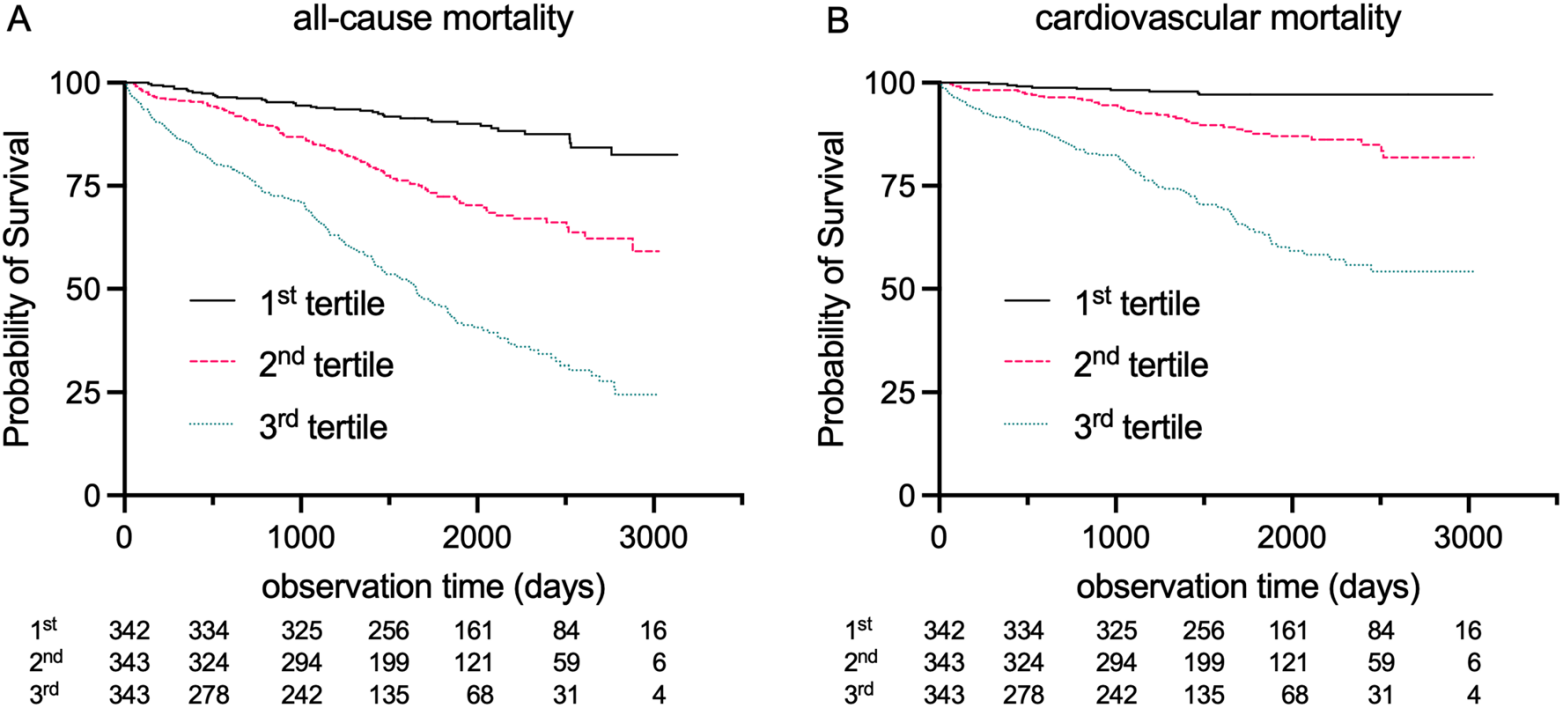
KM-graphs for tertiles of NT-proBNP. (**A**) Event rates for higher NT-proBNP tertiles were significantly higher for all-cause mortality (p < 0.001). (**B**) Similar results were seen for CV-mortality (p < 0.001).

During the observation period with a median of 4.6 years (25th percentile 3.2 years, 75th percentile 6.2) a total of 336 deaths were registered (overall event rate 32.7%, calculated event rate per year 7.1%). Furthermore, a total of 157 events were classified as CV-deaths (overall CV-death rate 15.3%, calculated event rate per year 3.3%). A clear-cut significant association (log-rank p < 0.001) between tertiles of NT-proBNP and both all-cause and CV-death was seen in Kaplan–Meier curves with the highest event rates in the highest NT-proBNP tertile (Fig. 2).

For further analyses, Cox-regression analyses for a continuous increase of one unit logNT-proBNP showed a significant association with all-cause death in crude regression analysis (hazard ratio 1.91, 95% confidence interval 1.77–2.07) and a multivariable-adjusted model (age, sex, diabetes mellitus, LDL-C, arterial hypertension, active smoking, eGFR, history of heart failure, Fontaine stage and BMI) for traditional CV risk factors and heart failure (HR 1.68, 95% CI 1.51–1.87). Likewise, results for CV death showed a significant association in a multivariable-adjusted model (HR 1.86, 95% CI 1.55–2.15).

Similar associations were found in patients without known heart failure (crude HR 1.94, 95% CI 1.77–2.13; multivariable-adjusted HR 1.65, 95% CI 1.47–1.86) as well as those with known heart failure (crude HR 1.92, 95% CI 1.55–2.38, multivariable-adjusted HR 2.03, 95% CI 1.49–2.77). Regarding CV-mortality associations remained significant both for sub analyses of known heart failure (HR 1.88, 95% CI 1.23–2.77) and without known heart failure (HR 1.83, 95% CI 1.52–2.21).

Likewise, significant associations were found in patients with IC (crude HR 1.79, 95% CI 1.61–2.00, multivariable-adjusted HR 1.62, 95% CI 1.42–1.86). Hazard ratios were even higher in patients with CLI (n = 212/252, crude HR 1.82, 95% CI 1.59–2.08, multivariable-adjusted HR 1.79, 95% CI 1.50–2.14). Analyses for CV mortality showed similar associations (IC HR 1.71, 95% CI 1.37–2.13; CLI HR 1.95, 95% CI 1.52–2.51).

A further sub analysis for PAD patients without other known CV comorbidities (excluding known myocardial infarction, known coronary artery disease, and previous stroke) revealed similar results (crude HR 1.94, 95% CI 1.72–2.19, multivariable-adjusted HR 1.69, 95% CI 1.44–1.99). The strongest HR was found in a sub analysis for CV mortality (HR 2.22, 95% CI 1.65–3.00) An overview of the results is shown in Table 3.

**Table 3:** Cox-Regression analyses for all-cause and CV death for an increase of one-unit logNT-proBNP.

	n	Cox regression analyses		
		Crude	Multivariable adjusted	
			All-cause mortality	CV-mortality
Overall	1028	**1.91 (1.77–2.07)**	**1.68 (1.51–1.87)**	**1.86 (1.55–2.15)**
Subanalyses				
History of heart failure	108	**1.92 (1.55–2.38)**	**2.03 (1.49–2.77)**	**1.88 (1.23–2.77)**
Without history of heart failure	920	**1.94 (1.77–2.13)**	**1.65 (1.47–1.86)**	**1.83 (1.52–2.21)**
Intermittent claudication	776	**1.79 (1.61–2.00)**	**1.62 (1.42–1.86)**	**1.71 (1.37–2.13)**
Critical limb ischemia	252	**1.82 (1.59–2.08)**	**1.79 (1.50–2.14)**	**1.95 (1.52–2.51)**
Without CV comorbidities	664	**1.94 (1.72–2.19)**	**1.69 (1.44–1.99)**	**2.22 (1.65–3.00)**

Data are presented as Hazard ratio and 95% confidence interval. Models were adjusted for age, sex, diabetes mellitus, LDL-C, arterial hypertension, active smoking, eGFR, history of heart failure, Fontaine stage and BMI. Bold letters show significant results. For the subanalysis of history of heart failure and without history of heart failure in multivariable models the co-variable history of heart failure was excluded from the regression models. Similarly, Fontaine stages were not included in subanalyses of intermittent claudication and critical limb ischemia.

**Figure 3:**
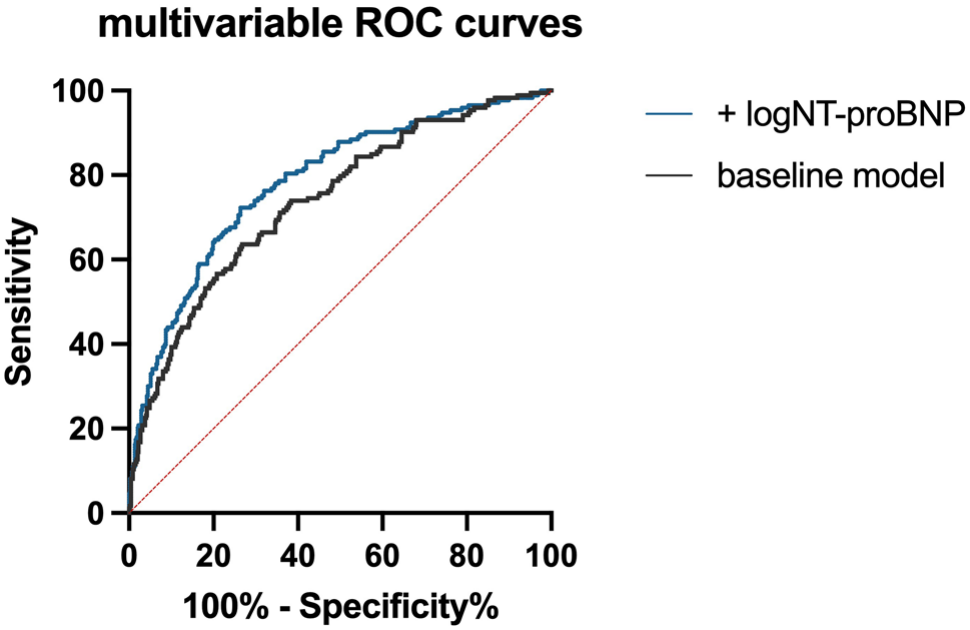
ROC graphs for the predictive power of all-cause mortality for addition of NT-proBNP. The AUC improved from 0.75 (0.71–0.78) in the baseline model (age, sex, diabetes mellitus, LDL-C, arterial hypertension, active smoking, eGFR, history of heart failure, Fontaine stage and BMI) with the addition of NT-proBNP 0.80 (0.77–0.83).

With Youden calculation a theoretically optimal cut-off point regarding all-cause mortality with a specificity of 67.8% and sensitivity of 73.1% for NT-proBNP was determined at 212.1 pg/ml. Furthermore, the added predictive power of NT-proBNP was calculated in receiver operating curve (ROC) analyses. The area under the curve (AUC) for the same multivariable model as used in the Cox-regression analyses increased from 0.75 (0.71–0.78) to 0.80 (0.77–0.83). with addition of NT-proBNP (Fig. 3).

## Discussion

This study has two major novel findings. Firstly, NT-proBNP levels are independently associated with the target lesion site for endovascular repair. Secondly, this study shows for the first time that NT-proBNP levels are associated with all-cause and CV mortality in patients with symptomatic PAD planned for endovascular repair. All regression analyses withheld multivariable adjustment for traditional risk factors including age. Moreover, a theoretical cut-off point for further extensive workup of elevated NT-proBNP levels was found at 212.2 pg/ml, which is below any age-adjusted cut-off value for chronic stable heart failure of most reported cut-off points, according to a large meta-analysis^17^. However, the recently updated guidelines on heart failure suggest echocardiography in any patient with a NT-proBNP > 125 pg/ml^15^. The ROC showed a satisfactory improvement for mortality in the multivariable model with the addition of NT-proBNP.

In one of the most recent outcome trials with a distinct PAD subcohort, FOURIER, LDL-C control with evolocumab led to an absolute risk reduction of remarkable 3.5%^3^. Similarly, remarkable absolute risk reductions were found in PAD sub analyses for CV outcome trials with GLP-1 RA^18^. However, contrary to primarily CV event reductions of GLP-1 RA and PCSK9i trials, dedicated and strong risk reduction rates were found in SGLT2i CV outcome trials with primarily driven results due to improvement of heart failure outcomes^19^.

Yet, no dedicated information on the combination of heart failure and PAD co-prevalence is available. One RCT showed a prevalence of self-reported known heart failure at 10%^6^, while several other large RCTs in PAD did not adjust their data for presence of heart failure or state prevalence of heart failure^20,21^. Thus, underreporting and/or undiagnosed HF might be a common problem, as we hypothesized.

NT-proBNP offers a feasible and abundantly examined biomarker for HF diagnosis^22–24^. Yet, results for NT-proBNP guided therapy are varying^25–27^. Despite influence of renal function or obesity, NT-proBNP was nevertheless found to be a robust marker of outcome prediction in several heart failure studies^28,29^. Furthermore, proBNP predicted outcome in presumably healthy study participants without known HF^12,30^, while NT-proBNP was found to be suboptimal for HF detection in the general population^31^. No definitive information on NT-proBNP levels for HF detection and outcome in symptomatic PAD is available.

Patients with PAD feature a highly similar risk profile with heart failure with reduced ejection fraction and heart failure with preserved ejection fraction. This can be seen with rates for CAD of about 25% according to the REACH registry^2^ and similar rates in our cohort. Diabetes mellitus is prevalent in more than 48% of patients with PAD, when extensively screened for this comorbidity^32^. Another detrimental contributor to a possible high rate of co-prevalent HF, apart from typically high rates of modifiable traditional CV risk factors, is the higher age of patients with PAD^33^. Our patients feature comparable risk factor rates to other dedicated PAD cohorts^32,34^ and thus might be generalizable to other typical PAD cohorts.

Previous cohorts evaluated NT-proBNP and PAD for outcome in a small sample size^35,36^ and in a cohort with an inclusion time of more than 20 years^37^. The mentioned study by Müller et al.^36^ however did not evaluate CV death and only ¾ of patients evaluated were eventually revascularized. Furthermore, only about half of the patients received currently suggested best medical treatment as only about 50% were on lipid lowering therapy – a cornerstone of PAD therapy. Yet, this study shows data for PAD patients, who were specifically admitted to an inpatient ward for endovascular repair of IC or CLI and featured high rates of CV secondary prevention therapy, which can be seen in statin intake rates of above 90% and similar treatment rates for RAAS blockade. We thus hypothesize that a major proportion of the high residual risk for mortality in PAD patients might be explained by high rates of undiagnosed HF. This hypothesis can be further argued with the shown subanalysis in which hazard ratios for patients without known HF and with known HF are similar. However, due to the study design no causative role of heart failure can be deducted. Thus, the association shown between mortality data and NT-proBNP levels can only be seen as interpretative. The current HF guidelines^38^ recommend further evaluation with echocardiography with an NT-proBNP > 125 pg/mL, however at the time of analysis this was not standard of care. Yet, a dedicated analysis on HF in patients with planned endovascular revascularization would further elucidate this issue.

Our finding of an association of BTK and multivessel target lesions with higher NT-proBNP levels was independent of traditional CV risk factors and the PAD Fontaine stage. In line with this finding are the higher levels of NT-proBNP in Fontaine stage IV patients, which was previously described in PAD^39^. The lower levels of NT-proBNP in the multisite lesion group in comparison to the BTK group could be explained with the small proportion of patients without BTK lesions in the multisite lesion group. We hypothesize that higher NT-proBNP levels might be associated with those lesions due to the reduced outflow caused by increased rates of HF and otherwise not relevant BTK lesions. Thus, HF might add the relevant further risk for wounds and CLI in selected PAD subgroups.

This study has several limitations. Firstly, no information on left ventricular ejection fraction or strain was obtained with echocardiography or magnetic-resonance tomography. Secondly, patients were assessed at a single center. Thirdly, no specific information on decompensation at NT-proBNP measurement is available. Fourthly, no specific information on non-fatal vascular events or hospitalization for HF during the observation period can be presented due to the study design.

However, several strengths have to be considered. Firstly, all patients were evaluated for PAD in a tertiary care center. Secondly, sample sizes for PAD are large and a large number of CLI patients were included. Thirdly, outcome data were evaluated after a long follow-up period and in patients with high treatment rates for secondary CV prevention.

In conclusion, this study evaluated NT-proBNP levels in patients with symptomatic PAD admitted for endovascular repair. NT pro-BNP was analyzed for outcome analysis and utilized as a surrogate biomarker of heart failure. Results showed that NT-proBNP levels were highly and independently associated with all-cause and CV-mortality in symptomatic PAD patients. Furthermore, an independent association of NT-proBNP was found with below-the-knee or multisite target lesions, which could further highlight patients at especially increased risk. A high rate of undiagnosed heart failure might be accountable for these findings. However, confirmatory dedicated analyses on heart failure and PAD, especially with a focus on major adverse cardiovascular events (MACE), are warranted.

## Methods

### Patients and study design

Patients of this cohort have been previously described^40^. In brief, patients of the Vienna Lip-LEAD study were retrospectively included in this study to evaluate traditional risk factor goals in a single-center study. The study was approved by the institutional ethics committee (Ethikkommission der Medizinischen Universität Wien) and follows the Declaration of Helsinki^41^ and contemporary institutional Good Clinical Practice guidelines. All patients that were admitted to the inpatient ward of the Division of Angiology, Medical University of Vienna, for endovascular repair of symptomatic PAD were included. Inclusion was performed retrospectively from 1/Jan/2013 to 31/Dec/2018. Informed consent was waived by the institutional review board (IRB—Ethikkommission der Medizinischen Universität Wien). In case of further admissions of individual patients during this time, only the first admission was implemented into this analysis.

Patients with missing co-variables, an eGFR < 15 ml/min or renal replacement therapy, prior organ transplantation, and known atrial fibrillation (AF) were excluded from further analysis (see supplemental Fig. 1).

Concomitant diseases, including coronary artery disease, history of myocardial infarction, history of stroke, history of previous PAD operations/endovascular repair, cerebrovascular diseases/carotid stenosis, diabetes mellitus, arterial hypertension, smoking history, and heart failure, were assessed by patient’s declaration and available medical records.

Laboratory parameters were routinely measured on the day of admission, including standard parameters of kidney and liver function and total blood cell count and inflammatory markers (c-reactive protein). NT-proBNP was measured on an (Roche Diagnostics platform) at the central laboratory of the General Hospital of Vienna directly after the blood draw.

Statin doses were harmonized with atorvastatin equivalency doses for better statistical comparison according to Naci et al.^42^. The CKD-EPI formula of 2012 was used for calculation^43^.

### PAD severity and lesion site

Symptomatic PAD and the need for revascularization were assessed by vascular consultants. PAD severity was clinically defined with the Fontaine staging system. Oscillometry and ABImeasurements were performed by specially trained vascular medical staff with a Doppler sonographic assessment (ELCAT, Wolfratshausen, Germany). ABI, as defined by the ratio of the brachial and ankle systolic blood pressure, was determined according to the TASC II criteria^44^. A resting ABI < 0.9 and a TBI < 0.7 was defined as pathological. Mediasclerosis was assumed in the case of incompressible arteries or an ABI > 1.4. Stenosis location and severity before endovascular repair were evaluated by ultrasonography or contrast-enhanced CT/MR-scans at clinicians’ discretion.

Angiography reports were reviewed by AF, AH, and BZ to ascertain the actual site of lesion repair. Lesion sites were subjected to four categories: 1—iliac region including kissing stenting of the common iliac artery; 2—femoral (AFS) and popliteal regions; 3—below the knee repair (BTK); 4—multiple regions (multisite).

### Outcome analysis

Outcome analyses were obtained from the central death database of the federal republic of Austria. Information on mortality was gathered until 31/Aug/2021. CV death was defined by a death diagnosis of the ICD I category.

### Statistical analysis

The entire statistical analysis was performed with SPSS 28.0 (SPSS Inc. Chicago, IL, USA). Figures were drawn with GraphPad Prism 9.0 (GraphPad Software Inc., San Diego, CA, USA). Data are presented as mean ± standard deviation (SD) or median and percentiles (25th, 75th), as appropriate. A two-sided p-value < 0.05 was defined as statistically significant. Student’s t-tests or nonparametric equivalents and chi-square tests were applied, as appropriate. NT-proBNP levels were divided into tertiles for graphical comparison and evaluated by Kaplan–Meier analyses. A log-rank test was used for statistical comparison. Cox regression analyses were performed with continuous analyses for an increase of one unit of logarithmically transformed NT-proBNP. A multivariable-adjusted model was performed with traditional CV risk factors (age, sex, diabetes mellitus, LDL-C, arterial hypertension, active smoking, eGFR, history of heart failure, Fontaine stage, and BMI). Relevant subgroups were analyzed with the same multivariable models. In addition, for the subanalysis of history of heart failure and without history of heart failure in multivariable models the co-variable history of heart failure was excluded from the regression models. Similarly, Fontaine stages were not included in subanalyses of intermittent claudication and critical limb ischemia. A theoretically optimal cut-off point for all-cause death was calculated according to the Youden method and ROC curves were fitted for model comparison between a multivariable model with and without NT-proBNP. NT-proBNP exhibited a skewed distribution and thus the logarithm was used throughout the manuscript.

## Supplementary Information

Supplementary Figure 1.

## Data Availability

Data are available from the corresponding author upon reasonable request.
