# Supplementary figures and images for: NT-proBNP as a surrogate for unknown heart failure and its predictive power for peripheral artery disease outcome and phenotype
Source: Sci Rep. 2023 May 17;13:8029. doi: 10.1038/s41598-023-35073-z (PMC10192354; doi:10.1038/s41598-023-35073-z)

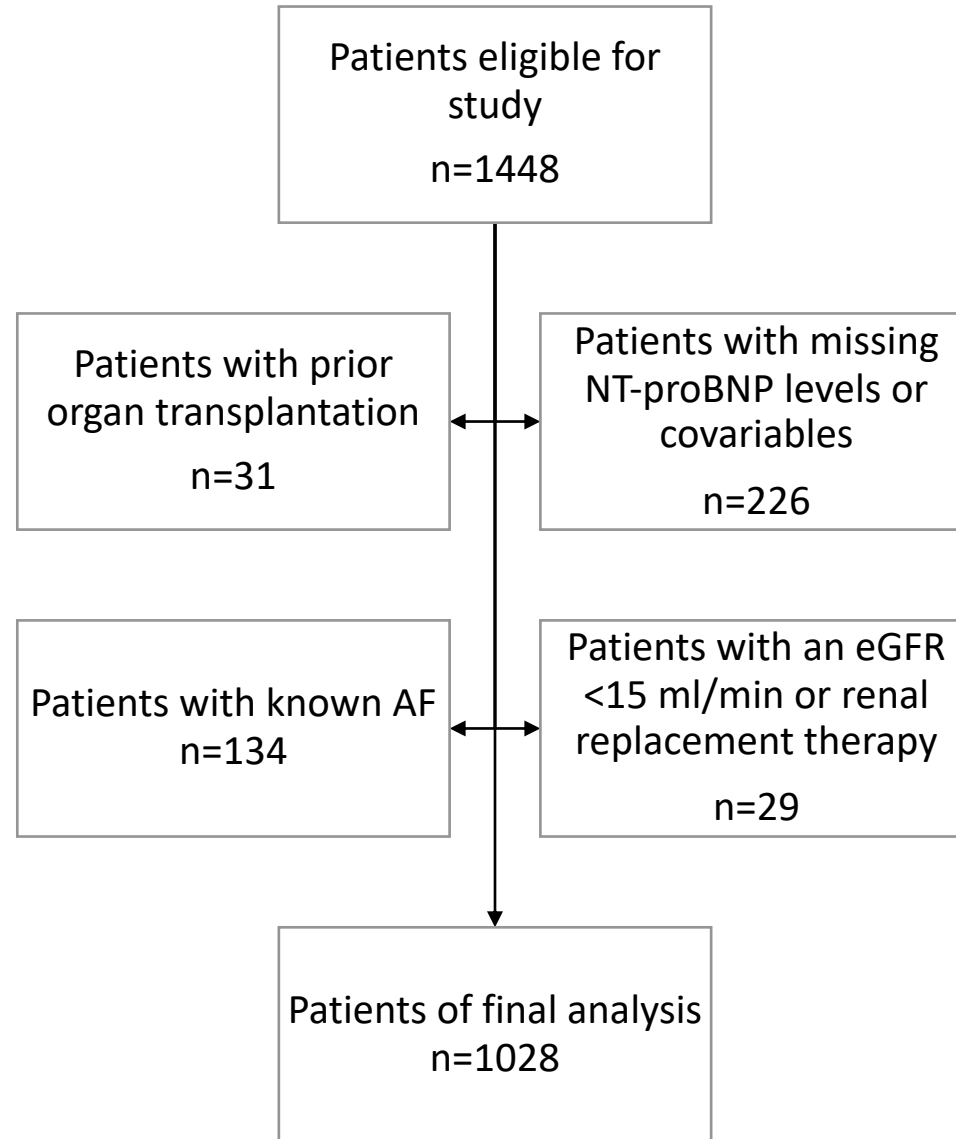

Supplemental Figure 1: Flow chart of patient selection

Supplement: Supplementary file 1 — Supplementary Figure 1. [file 41598_2023_35073_MOESM1_ESM.pdf]
